# Gut microbiota-based biomarkers for precision subtype classification and mechanistic understanding of biliary and hyperlipidemic acute pancreatitis

**DOI:** 10.3389/fmicb.2025.1695811

**Published:** 2025-11-18

**Authors:** Xinyu Deng, Xueqian Wu, Ruobing Wang, Xiaohan Qiao, Ting Cao, Yao Xu, Qun Jin, Lingling Jia, Wei Liang

**Affiliations:** 1 Department of Laboratory Medicine, The First Affiliated Hospital of Ningbo University, Ningbo, Zhejiang, China; 2 College of Food Science and Engineering, Ningbo University, Ningbo, Zhejiang, China; 3 Joint Logistics Support Force No. 906 Hospital, Ningbo, Zhejiang, China; 4 Zhejiang Engineering Research Center of Innovative Technologies and Diagnostic and Therapeutic Equipment for Urinary System Diseases, Ningbo, Zhejiang, China

**Keywords:** biliary pancreatitis, hyperlipidemic pancreatitis, gut microbiome, host-microbiome interaction, biomarkers

## Abstract

**Background:**

Acute pancreatitis (AP) is an inflammatory disorder with distinct etiological subtypes, yet the role of gut microbiota in disease pathogenesis remains poorly understood. We hypothesized that biliary acute pancreatitis (BAP) and hyperlipidemic acute pancreatitis (HLAP) exhibit etiology-specific gut microbiota signatures that correlate with disease severity and metabolic dysfunction.

**Methods:**

We conducted a cross-sectional study in which stool samples were collected from 20 BAP patients, 20 HLAP patients, and 20 healthy controls (HC) for 16S rRNA gene sequencing to compare gut microbiota profiles among the three groups. Microbial diversity, taxonomy, and functional genes were analyzed using bioinformatics pipelines. Clinical-microbial correlations were assessed, and the construction of RF and logistic regression models evaluated diagnostic biomarker potential.

**Results:**

Both AP groups showed significantly reduced microbial diversity compared to controls, with HLAP patients exhibiting more severe dysbiosis. HLAP patients showed enrichment of pro-inflammatory taxa, including *Escherichia-Shigella* and *Collinsella*, alongside depletion of beneficial genera *Faecalibacterium* and *Bifidobacterium*. As a key SCFA-producing genus, *Faecalibacterium* exhibited comprehensive correlations with inflammatory markers, pancreatic enzymes, and lipid profiles in Spearman correlation analysis. Functional analysis revealed compromised short-chain fatty acid biosynthesis capacity, as evidenced by significant downregulation of acetate (*ackA*, *pta*) and butyrate (*buk*, *but*) synthesis genes in AP patients, which may have partially mediated the observed differences in microbiota composition. Furthermore, our findings reveal that multi-species biomarker panels provide superior diagnostic performance compared to single-species predictors for BAP and HLAP subtype classification.

**Conclusion:**

BAP and HLAP patients exhibit distinct gut microbiota signatures with progressive dysbiosis, functional impairment, and strong host associations. These findings establish a novel framework linking gut microbial composition to AP pathophysiology, providing insights for microbiome-targeted precision medicine strategies.

## 1 Introduction

Acute pancreatitis (AP) is a disease characterized by acute inflammatory responses in the pancreas with distinct etiological subtypes that differ significantly in pathogenesis, clinical presentation, and prognosis (Boxhoorn et al., 2020). Among various etiologies, biliary acute pancreatitis (BAP) remains the leading cause of AP (Hamada et al., 2020). BAP results from gallstone migration, causing duct obstruction at the bile duct, pancreatic duct, or both. The resulting increased duct pressure promotes pancreatitis through unregulated activation of digestive enzymes (van Geenen et al., 2010). With improved living standards and dietary changes in China, HLAP has emerged as the second leading cause of AP, surpassing alcohol in several regions, including Beijing and southern Sichuan (Wu et al., 2022; Zheng et al., 2015). The pathogenesis of HLAP primarily involves lipotoxic mechanisms where elevated triglycerides lead to free fatty acid accumulation in pancreatic microcirculation, causing local ischemia and inflammatory cascades (De Pretis et al., 2020). This lipid-mediated pancreatic injury pathway differs fundamentally from the mechanical obstruction seen in BAP. Accumulating evidence indicates that HLAP patients demonstrate higher rates of infected pancreatic necrosis (IPN), organ failure, prolonged hospitalization, and increased mortality (Pascual et al., 2019; Nawaz et al., 2015). These fundamental pathogenic and clinical differences between BAP and HLAP suggest that targeted research on both subtypes is essential for advancing AP management.

The gut-pancreas axis concept has provided novel insights into AP pathophysiology (Zhou et al., 2024; Zhang et al., 2022). Previous studies have identified the intestine as the organ most susceptible to injury during pancreatitis (Leveau et al., 2005). The resulting intestinal barrier dysfunction provides conditions for bacterial translocation, which worsens the original injury to the pancreas and triggers systemic inflammatory responses (Sun et al., 2024). This inflammatory environment promotes microbial dysbiosis, characterized by reduced diversity, beneficial bacteria depletion, and pathogenic taxa enrichment (van den Berg et al., 2021; Wu et al., 2023). Given the fundamental pathogenic differences between BAP and HLAP, these two subtypes likely exhibit distinct gut microbiota profiles, which could advance understanding of AP pathophysiology and enable precision diagnostics. However, comparative analyses of microbial signatures and their mechanistic roles in AP remain largely unexplored.

Therefore, this study aims to characterize gut microbiota composition among patients with BAP, HLAP, and healthy controls, and to elucidate how etiology-specific host factors shape distinct microbial signatures. Building on these insights, we sought to explore the microbial and metabolic mechanisms underlying gut-pancreas crosstalk and its disruption in AP. Ultimately, to translate microbiota-derived findings into potential clinical applications, we focused on identifying key microbial biomarkers and developing diagnostic models for precision AP subtype classification.

## 2 Materials and methods

### 2.1 Study design

**Figure 1:**
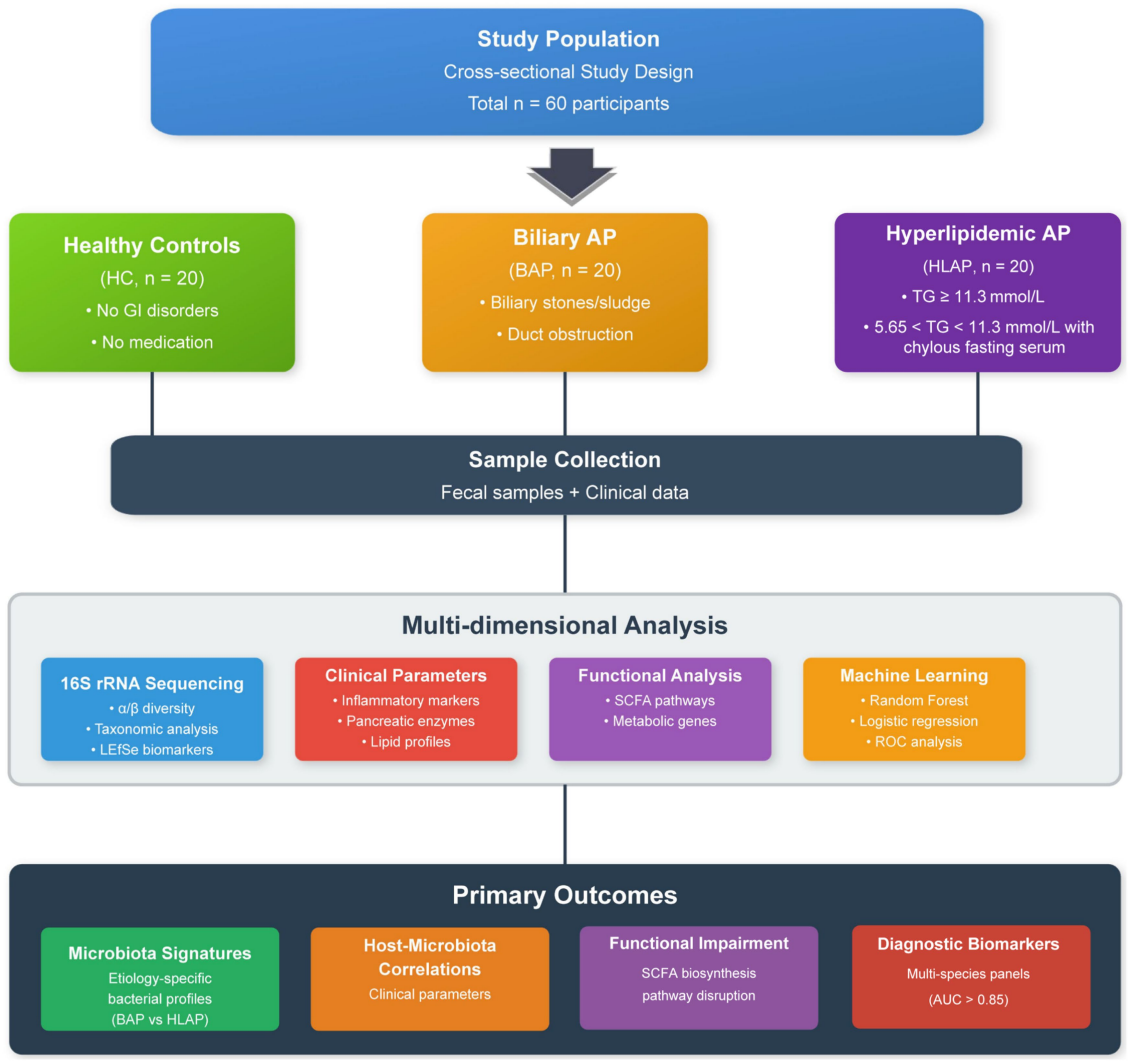
Flow diagram of this study.

The flow diagram of this study is shown in Figure 1.

### 2.2 Study participants and grouping

A total of 60 participants were enrolled in this study, including 20 patients with biliary acute pancreatitis (BAP), 20 patients with hyperlipidemic acute pancreatitis (HLAP), and 20 healthy controls (HC). All patients were diagnosed with acute pancreatitis at the Department of Gastroenterology, Affiliated Hospital of Ningbo University, based on the 2012 revised Atlanta criteria (Banks et al., 2013). The BAP diagnostic criteria were as follows: (1) having gallstones confirmed by abdominal ultrasound, CT, MRCP or other imaging examination; (2) having two or more of the following laboratory examination indicators: ① alkaline phosphatase (AKP) > 125 U/L, ② alanine transaminase (ALT) > 150 U/L, ③ total bilirubin (TBIL) > 2.3 mg/dL, and ④ gamma-glutamyl transferase (GGT) > 40 U/L (Zver et al., 2022). HLAP was defined as serum triglycerides ≥ 11.3 mmol/L or the serum TG levels between 5.65 to 11.3 mmol/L accompanied by chylous fasting serum without other etiologies of AP (Li et al., 2023; Tenner et al., 2024). Exclusion criteria included: (1) concurrent gastrointestinal diseases other than AP; (2) recent antibiotic use within the past month; and (3) the presence of severe comorbidities preventing proper sample collection or clinical assessment. Written informed consent was obtained from all participants, and clinical data were anonymized before analysis. This study was approved by the Ethics Committee of The First Affiliated Hospital of Ningbo University (No. 2025138A; 28 May 2025), and conducted in accordance with the Declaration of Helsinki.

### 2.3 Clinical data collection

Relevant clinical data of AP patients, including routine blood test results, serum amylase, and lipase levels, were extracted from the hospital’s Electronic Medical Record (EMR) system. The clinical laboratory tests were performed on the same day as stool collection. Healthy control data were obtained during physical examinations.

### 2.4 Fecal sample collection

For AP patients, approximately 1 g of stool was collected using sterile cryotubes on the first day of hospital admission. Samples were immediately packed on ice and transported to the laboratory from the Affiliated Hospital of Ningbo University. Upon arrival, fecal samples were divided into three aliquots and stored at −80 °C until further analysis. Stool from HC was collected during routine physical examinations using the same protocol to ensure consistency.

### 2.5 16S rRNA gene sequencing

Microbial genomic DNA was extracted from approximately 200 mg of fecal material using the QIAamp Fast DNA Stool Mini Kit following the manufacturer’s instructions. The V3-V4 hypervariable regions of the bacterial 16S rRNA gene were amplified using universal primers 341F and 806R. PCR products were purified, quantified, and sequenced using the Illumina NovaSeq 6,000 platform (paired-end 250 bp reads).

### 2.6 Real-time qPCR

The total fecal microbial DNA was obtained through the Fecal Genome DNA Extraction Kit (AU46111-96, BioTeke, China) according to the standard procedure of the manufacturer. The concentration and quality of DNA were assessed using a NanoDrop ND-1000 spectrophotometer (Thermo Fisher Scientific, Waltham, MA, United States). The SuperStar Universal SYBR Master Mix kits (Cowin Biotech, China) were used to determine the DNA levels of acetate kinase A (*ackA*), phosphotransacetylase (*pta*), butyrate kinase (*buk*), and butyryl-CoA (*but*). Calculations were conducted based on the comparative cycle threshold method (2^−∆∆Ct^). The primers used in this study are provided in Table 1.

**Table 1:** Primer sequences used for RT-qPCR analysis.

Gene name	Primer sequence	
	Forward	Reverse
Total bacteria	TCCTACGGGAGGCAGCAGT	GACTACCAGGGTATCTAATCCTGTT
Acetate kinase A	CAAACTGCTGACCAAAGAGT	GCGGTAGTTGTCTTCAACAT
Phosphotransacetylase	AACTGAACGCACCGGTTGAT	GAAGAGTCGTCGAAAATCTC
Butyrate kinase	CCATGCGTTAAACCAAAAAGC	AATACCTCCGCCCATATG
Butyryl-coenzyme A	GCIGAICATTTCACITGGAAYWSITGGCAYATG	CCTGCCTTTGCAATRTCIACRAANG

### 2.7 Statistical analysis

The data were analyzed using SPSS 25.0 statistical software (IBM, USA). Continuous variables with normal distribution were presented as the mean ± standard deviation (SD), and Statistical analysis among multiple groups was performed using one-way ANOVA. Continuous variables with non-normal distribution were presented as the median (P25, P75), and statistical analysis among multiple groups was performed using the Kruskal-Wallis test. Categorical data were expressed as percentages (%), and comparisons between groups were performed using a *χ*^2^ test. Multivariate logistic regression analysis was used to determine the independent predictors of PSD. Raw reads were processed using the QIIME2 pipeline or DADA2 for quality filtering, denoising, chimera removal, and sequence clustering into amplicon sequence variants (ASVs). Taxonomic annotation was performed using the SILVA 138 reference database (Lloyd-Price et al., 2019).

## 3 Results

### 3.1 Clinical characteristics of BAP, HLAP, and HC groups

As shown in Table 2, the demographic and clinical parameters were compared among the HC, BAP, and HLAP groups. There were no significant differences in gender composition across the three groups (*χ*^2^ = 0.600, *p* = 0.741), indicating that sex was not a confounding variable in subsequent analyses. However, the incidence of hypertension (HTN) was significantly higher in the HLAP group (55.0%) compared to the BAP (20.0%) and HC (10.0%) groups (*χ*^2^ = 10.999, *p* = 0.004). Additionally, fatty liver disease (FLD) also showed group-specific differences (*χ*^2^ = 7.267, *p* = 0.026). Serum amylase (AMY) and lipase (LPS), established diagnostic biomarkers of AP that reflect pancreatic acinar cell injury and enzyme release into systemic circulation, were elevated in both BAP and HLAP patients (Ross et al., 2021). In contrast, inflammatory markers (CRP and WBC) correlated more closely with disease severity (Farrell et al., 2021), with HLAP patients exhibiting the highest median values (*p* < 0.001), reflecting more severe inflammatory responses in this subtype. Moreover, lipid parameters exhibited significant alterations, with HLAP patients showing higher serum triglyceride (TG) levels (*p* < 0.001). These findings establish the clinical and biochemical foundation for understanding etiology-specific microbiota differences between BAP and HLAP. The elevated pancreatic enzymatic activity and inflammatory response observed in both AP subtypes reflect their systemic pathophysiological impact, while the divergent patterns in lipid metabolism and associated comorbidities (including FLD and HTN) may shape unique disease microenvironments, thereby contributing to the divergent gut microbiota profiles.

**Table 2:** Clinical characteristics and laboratory results among three groups.

Variables		HC	BAP	HLAP	F/H/χ^2^	*p*
Number		20	20	20		
Age (years)		31.50 (25.25, 39.25)	59.30 ± 16.41	47.70 ± 16.01	21.073	<0.001
BMI		22.27 ± 0.71	24.89 ± 3.52	25.54 ± 3.38	7.379	0.001
Gender, *n* (%)	Male	14 (70.0)	14 (70.0)	12 (40.0)	0.600	0.741
	Female	6 (30.0)	6 (30.0)	8 (60.0)		
HTN, *n* (%)		2 (10.0)	4 (20.0)	11 (55.0)	10.999	0.004
DM, *n* (%)		0 (0.0)	4 (20.0)	3 (15.0)	4.205	0.122
FLD, *n* (%)		1 (5.0)	4 (20.0)	8 (40.0)	7.267	0.026
CRP (mg/L)		<0.50	40.05 (8.80, 89.29)	49.99 (2.95, 146.10)	40.880	<0.001
WBC (*10^9^/L)		5.90 (5.10, 6.40)	7.50 (6.15, 8.75)	9.89 ± 4.16	15.013	<0.001
NE%		51.47 ± 6.77	74.97 ± 10.57	80.25 (59.03, 84.45)	30.144	<0.001
LY%		39.15 (32.88, 42.53)	15.90 ± 7.68	11.75 (6.33, 27.68)	33.038	<0.001
AMY (U/L)		75.5 (57.5, 78.8)	186.5 (77.5, 711.3)	145.5 (109.0, 345.5)	18.920	<0.001
LPS (U/L)		48.5 (32.5, 53.0)	164.5 (49.0, 566.3)	160.5 (94.5, 529.8)	20.840	<0.001
TG (mmol/L)		1.01 (0.80, 1.38)	1.03 (0.64, 2.17)	10.72 (6.85, 11.79)	36.293	<0.001
TC (mmol/L)		4.49 ± 0.73	3.80 (3.35, 4.49)	5.24 (3.60, 8.30)	5.380	0.068
HDL (mmol/L)		1.24 (1.09, 1.63)	0.99 (0.65, 1.07)	1.09 (0.91, 1.45)	10.610	0.005
LDL (mmol/L)		2.77 ± 0.66	2.35 (2.39, 3.31)	2.88 ± 1.38	1.055	0.590

BMI, body mass index; HTN, hypertension; DM, diabetes mellitus; FLD, fatty liver disease; CRP, C-reactive protein; WBC, white blood cell count; NE%, neutrophil percentage; LY%, lymphocyte percentage; AMY, amylase; LPS, lipase; TG, triglycerides; TC, total cholesterol; HDL, high-density lipoprotein cholesterol; LDL, low-density lipoprotein cholesterol.

### 3.2 Comprehensive analysis of gut microbiota diversity and community structure

**Figure 2:**
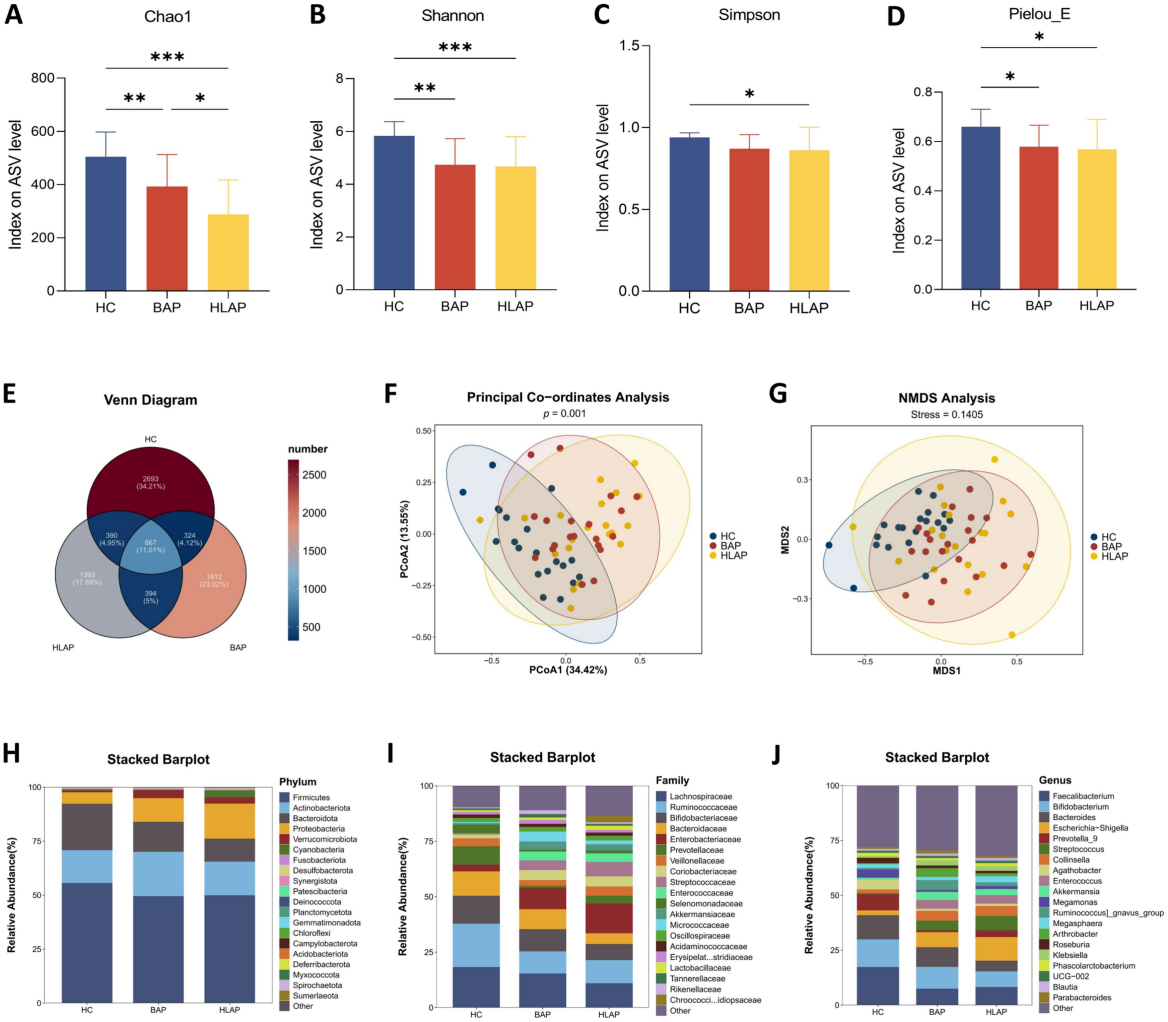
Changes of gut microbiota in the HC, BAP, and HLAP groups based on 16S rRNA data. *α*-diversity analysis showing that **(A)** the Chao1 index, **(B)** the Shannon index, **(C)** the Simpson index, and **(D)** the Pielou_E index were decreased in the BAP and HLAP groups. **(E)** Venn diagram of the observed ASVs in the HC, BAP, and HLAP groups. **(F)** Principal coordinate analysis. **(G)** NMDS analysis. **(H–J)** The taxonomic composition among the groups at the phylum, family, and genus levels. Data are presented as mean ± SD (*n* = 20 per group). *p < 0.05, **p < 0.01, ***p < 0.001.

Microbial richness and diversity, as evaluated by the Chao1, Shannon, Simpson, and Pielou_E indices, were markedly decreased in both the BAP group and HLAP group, compared to the HC group (Figures 2A–D). A Venn diagram analysis revealed that the number of group-specific ASVs was highest in the HC group (*n* = 2,693), followed by the BAP group (*n* = 1812) and HLAP group (*n* = 1,393) (Figure 2E). These results indicate a progressive loss of microbial diversity and uniqueness from healthy individuals to AP patients, with the HLAP group exhibiting the most marked reduction in unique ASVs, suggesting a more severe disturbance of the gut microbial ecosystem. Principal coordinates analysis (PCoA) further revealed a clear separation in microbial community structure between the HC and AP groups (Figures 2F–G), indicating AP-induced dysbiosis. We next examined microbial community composition at the phylum (Figure 2H), family (Figure 2I), and genus (Figure 2J) levels. At the phylum level, Firmicutes, Actinobacteriota, and Bacteroidota dominated the microbial community composition. Consistent with previous reports, the enrichment of Proteobacteria, a common hallmark of microbiota dysbiosis (Reuvers et al., 2022), was more pronounced in the HLAP group compared to the BAP group. Notably, the top three most abundant families (Lachnospiraceae, Ruminococcaceae, and Bifidobacteriaceae) represent key SCFA-producing taxa, suggesting that gut-pancreas axis interactions may be primarily mediated through these metabolically active microbial communities (Sánchez-Tapia et al., 2020; Song et al., 2025). These findings prompted us to investigate whether the observed microbiota alterations translated into compromised SCFA biosynthetic capacity.

### 3.3 Differential taxonomic composition across multiple levels

**Figure 3:**
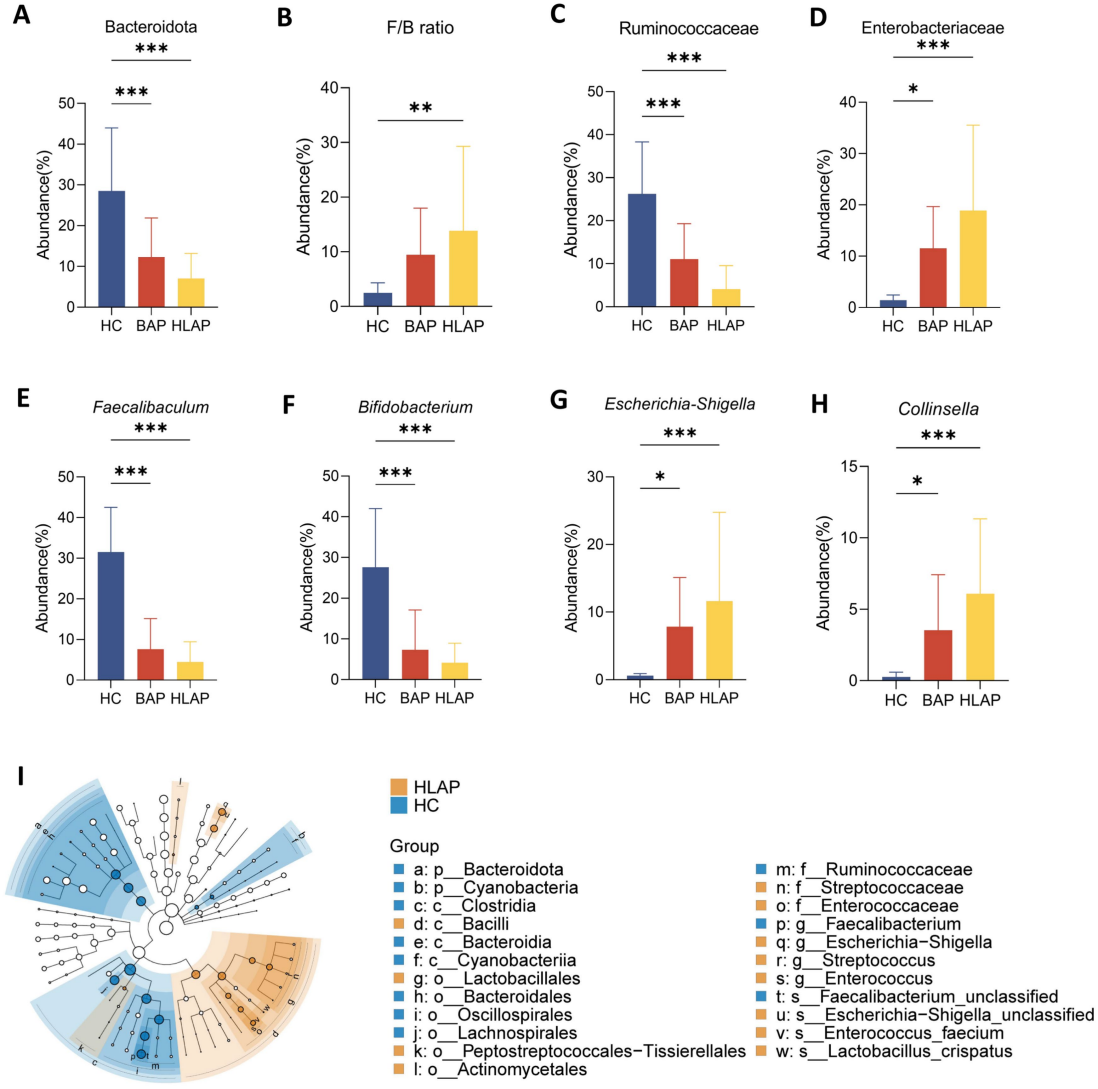
Taxonomic analysis of gut microbiota differences across multiple levels. **(A)** Relative abundance of Bacteroidota. **(B)** The Firmicutes/Bacteroidota (F/B) ratio. **(C,D)** Taxonomic analysis of gut microbiota differences at the family level (Ruminococcaceae and Enterobacteriaceae). **(E–H)** Taxonomic analysis of gut microbiota differences at genus level: **(E)** *Faecalibacterium*, **(F)** *Bifidobacterium*, **(G)** *Escherichia-Shigella*, and **(H)** *Collinsella*. **(I)** Differentially abundant taxa identified by LEfSe analysis (LDA score > 3). Data are presented as mean ± SD (*n* = 20 per group). *p < 0.05, **p < 0.01, ***p < 0.001.

At the phylum level, Firmicutes and Bacteroidota dominated across all groups. While Firmicutes showed no significant differences, Bacteroidota was markedly depleted in both AP groups vs. controls (Figure 3A). The Firmicutes/Bacteroidota (F/B) ratio serves as a crucial indicator of gut microbiota homeostasis, with elevated ratios typically associated with metabolic dysfunction, inflammation, and compromised intestinal barrier integrity (Houtman et al., 2022). As shown in Figure 3B, the F/B ratio was significantly elevated in the HLAP group compared to the HC group (*p* < 0.01), while the BAP group showed no significant difference from the HC group. At the family level, Enterobacteriaceae abundance was higher in HLAP (*p* < 0.001), whereas Ruminococcaceae were markedly decreased (*p* < 0.001) (Figures 3C,D). At the genus level, pro-inflammatory taxa *Escherichia-Shigella* and *Collinsella* were enriched in HLAP (*p* < 0.001), while beneficial genera *Faecalibacterium* and *Bifidobacterium* were significantly depleted (*p* < 0.001) (Figures 3E–H). LEfSe analysis revealed distinct microbial signatures among groups (Figure 3I). Key biomarkers included Bacteroidota and Cyanobacteria at the phylum level, Ruminococcaceae at the family level, and *Faecalibacterium* at the genus level. These findings motivated the establishment of predictive models to achieve precise AP subtype classification, thereby translating the observed microbiota differences into potential clinical utility.

### 3.4 Host-microbiota interaction networks and clinical correlations

**Figure 4:**
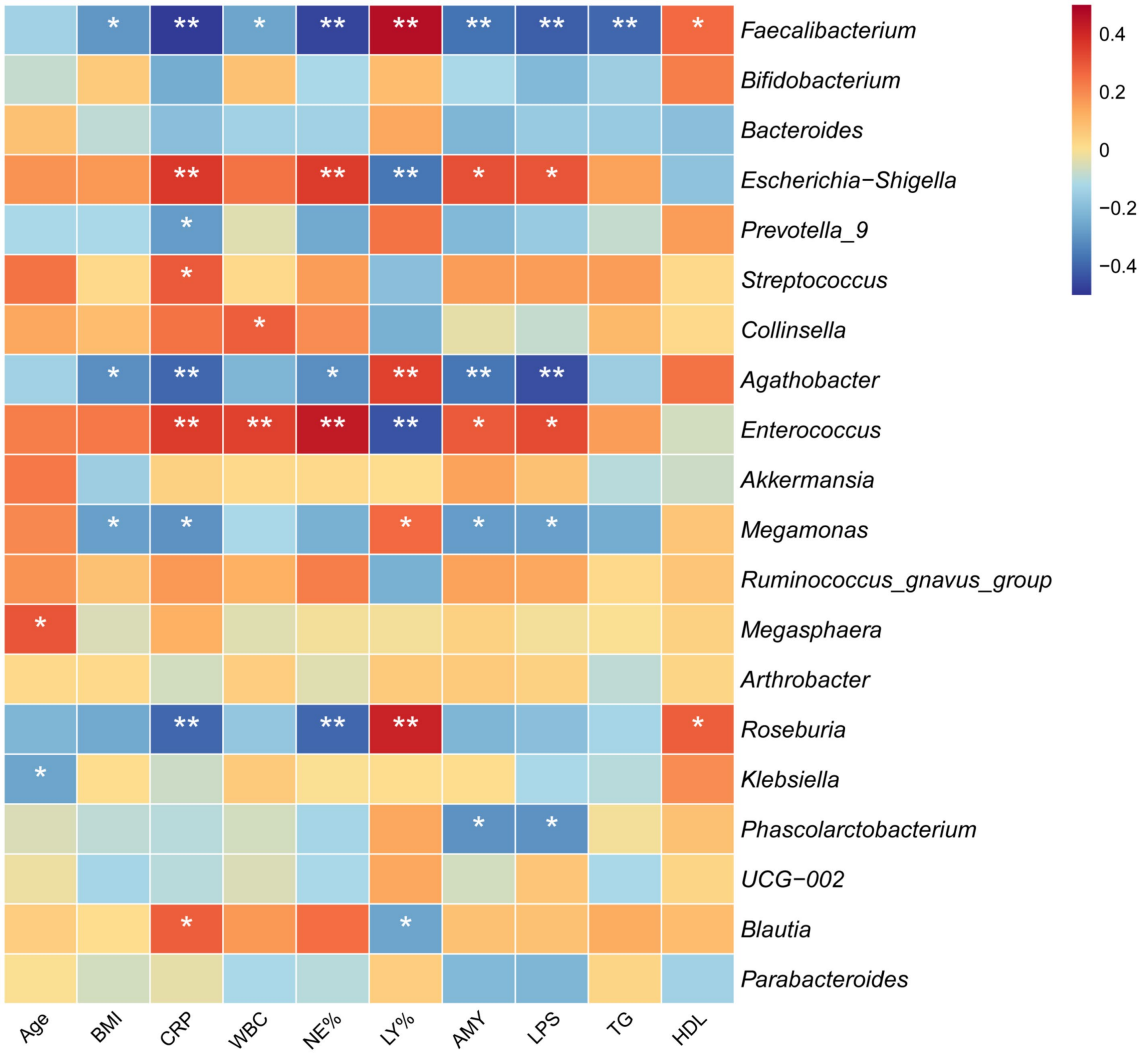
Heatmap of correlations between the top 20 most abundant genera and key clinical parameters in AP patients.

To explore the host-microbiome associations, we performed Spearman correlation between the top 20 abundant genera and 10 clinical indicators, which showed significant differences in Table 2 and Figure 4. *Faecalibacterium*, as the most abundant genus among the top 20, demonstrated comprehensive associations with inflammatory markers, pancreatic enzymes, and lipid metabolism parameters in correlation analysis. Notably, *Agathobacter*, despite displaying similar correlations with inflammatory and pancreatic markers, lacked associations with lipid profiles (TG, HDL). This suggests that different bacterial genera exhibit distinct regulatory responses in BAP versus HLAP patients, with metabolically active bacteria like *Faecalibacterium* (a key SCFA-producing genus) being more sensitive to lipid-associated pathological changes (Lopez-Siles et al., 2017). In contrast, the pro-inflammatory taxa *Escherichia-Shigella* and *Enterococcus* correlated positively with inflammatory markers (CRP, NE%), with *Escherichia-Shigella* enrichment in HLAP associated with more severe inflammation (van den Berg et al., 2021). *Megamonas* and *Phascolarctobacterium* are also negatively correlated with pancreatic enzymes (AMY, LPS), exhibiting their relevance as potential diagnostic markers in AP. Additionally, our analysis revealed that age did not show significant correlations with gut microbiota composition overall. Although age was correlated with *Megasphaera* and *Klebsiella*, these two genera showed no correlations with any of these clinical indicators examined, indicating minimal confounding from age differences among groups. Collectively, these findings suggest distinct associations between bacterial genera and AP-related inflammatory responses and lipid metabolism, highlighting potential mechanistic links.

### 3.5 Functional genes analysis of microbial metabolic pathways

**Figure 5:**
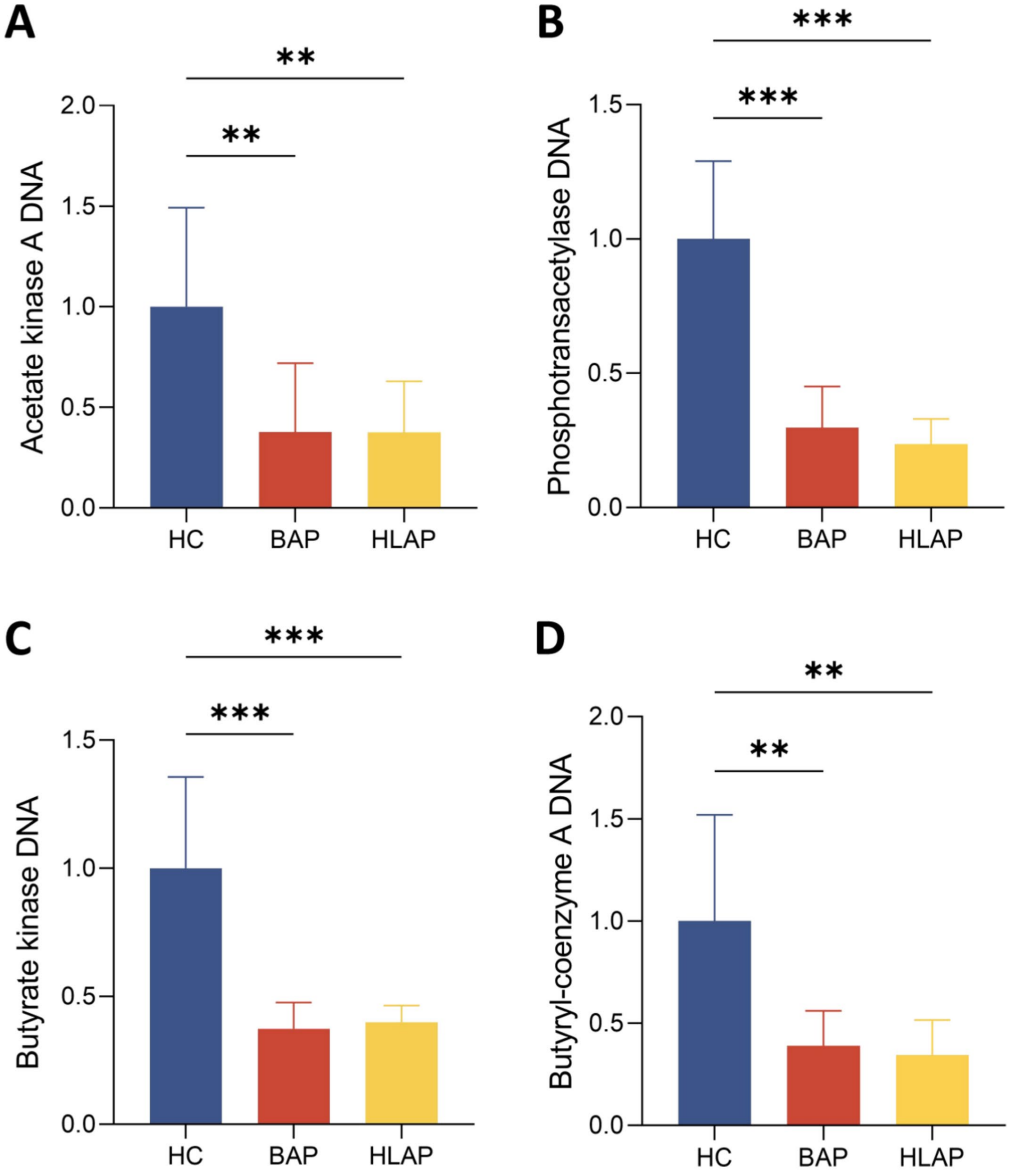
Relative expression levels of SCFA-related metabolic genes in fecal samples. **(A,B)** Acetate pathway: acetate kinase, phosphotransacetylase. **(C,D)** Butyrate pathway: butyrate kinase, butyryl-CoA. Data are presented as mean ± SD (*n* = 8 per group). *p < 0.05, **p < 0.01, ***p < 0.001.

Short-chain fatty acids (SCFAs), especially acetate and butyrate, are the main products of dietary fiber fermentation in the colon (Ikeda et al., 2022). Acetate production primarily involves two key genes, *ackA* and *pta*, encoding acetate kinase and phosphotransacetylase respectively, while gut microbes produce butyrate through two main pathways, the butyrate kinase pathway (*buk*) and the butyryl-CoA pathway (*but*) (De Mets et al., 2019; Gharechahi et al., 2021). Acetate serves as a key metabolic regulator that can suppress adipocyte lipolysis, thereby maintaining lipid homeostasis (May and den Hartigh, 2021). We observed a consistent downward trend in acetate synthesis genes *ackA* and *pta*, indicating compromised microbial acetate-generating potential in AP patients (Figures 5A,B). This impaired acetate production may exacerbate lipolysis and lipid dysregulation in HLAP patients (Lei et al., 2021). Major attention is focused on butyrate for its anti-inflammatory effects in AP (Xiong et al., 2022). Through stimulating MUC2 production and modulating tight junction protein expression, butyrate can reinforce intestinal barrier integrity and reduce LPS translocation, thereby attenuating inflammatory response in pancreatic tissues (Peng et al., 2024). Given these critical roles of butyrate, we investigated whether the microbiota alterations in AP patients translate into impaired butyrate biosynthesis capacity. Notably, the expression of the two key genes was markedly reduced in AP patients compared to the HC group (Figures 5C,D). Despite similar patterns of microbial metabolic impairment between AP subtypes, the pronounced SCFA biosynthetic deficiency observed in both BAP and HLAP groups highlights potential therapeutic targets for modulating gut microbiota function in AP management.

### 3.6 Clinical diagnostic value assessment and biomarker development

**Figure 6:**
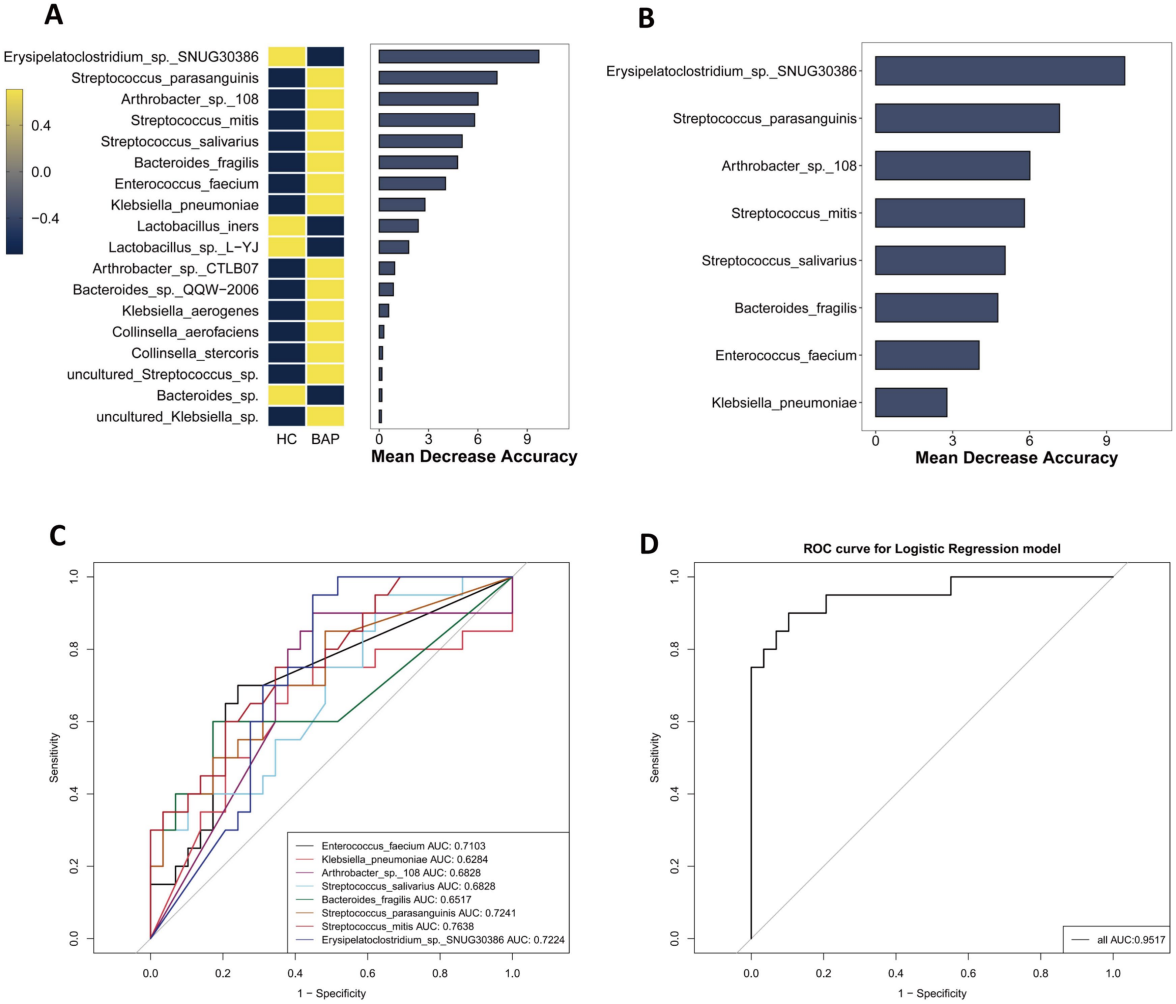
Gut microbiota-based classification model for BAP vs. HC discrimination. **(A)** Importance ranking of microbial biomarkers by random forest analysis (*n* = 20). **(B)** Relative abundance of top 8 bacterial biomarkers (*n* = 20). **(C)** Individual ROC curves for each of the top 8 biomarkers (*n* = 20). **(D)** Combined ROC curve for the multi-species classification model (*n* = 20).

**Figure 7:**
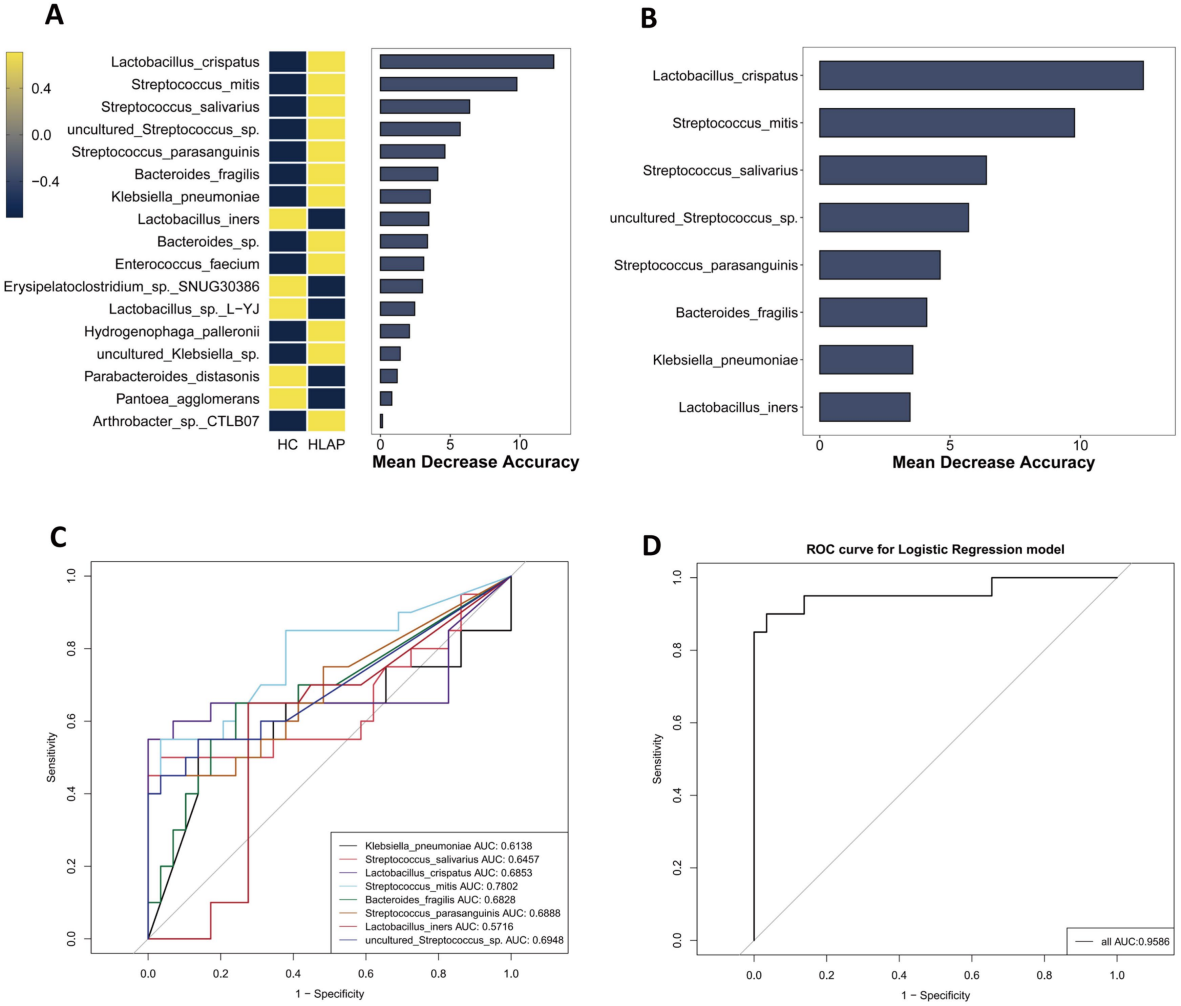
Gut microbiota-based classification model for HLAP vs. HC discrimination. **(A)** Importance ranking of microbial biomarkers by random forest analysis (*n* = 20). **(B)** Relative abundance of top 8 bacterial biomarkers (*n* = 20). **(C)** Individual ROC curves for each of the top 8 biomarkers (*n* = 20). **(D)** Combined ROC curve for the multi-species classification model (*n* = 20).

**Figure 8:**
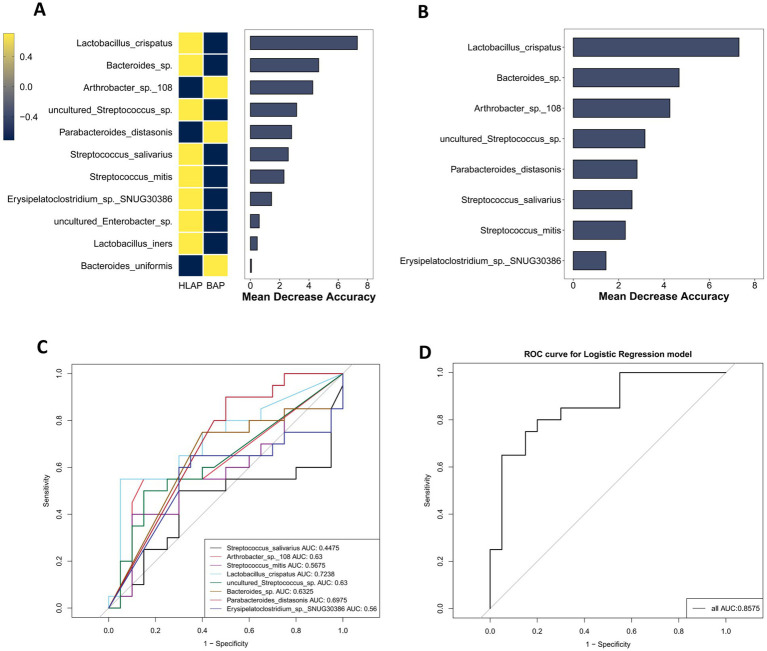
Gut microbiota-based classification model for BAP vs. HLAP discrimination. **(A)** Importance ranking of microbial biomarkers by random forest analysis (*n* = 20). **(B)** Relative abundance of top 8 bacterial biomarkers (*n* = 20). **(C)** Individual ROC curves for each of the top 8 biomarkers (*n* = 20). **(D)** Combined ROC curve for the multi-species classification model (*n* = 20).

To evaluate the clinical significance of the gut microbiota both in BAP and HLAP, this study constructed RF and logistic regression models, based on the relative abundances of microbial species (Zou et al., 2022). Through the filtration of species with abundances less than 1%, 18 taxa were identified as potential biomarkers for BAP, as indicated by their mean decrease accuracy (Figure 6A). To evaluate the discriminatory ability of these species between BAP patients and healthy controls, a classification model was constructed using the top 8 species (Figure 6B), and the accuracy of the model in predicting health status was assessed via ROC curves (Figure 6C). Among the single-species predictions, *Streptococcus mitis* demonstrated the highest individual predictive power (AUC = 0.7638), followed by *Streptococcus parasanguinis* (AUC = 0.7241). Notably, inclusion of all eight differentially abundant species markedly enhanced the predictive performance of the model (AUC = 0.9517, Figure 6D). Similarly, for HLAP classification, 17 taxa were identified as potential biomarkers, with the top 8 species used for model construction (Figures 7A,B). ROC curve analysis revealed comparable results, with the combined eight-species model achieving an AUC of 0.9586 (Figures 7C,D). Furthermore, we assessed the discriminatory capacity between BAP and HLAP subtypes (Figures 8A,D). *Lactobacillus crispatus* showed the strongest individual predictive capacity in single-species analysis (AUC = 0.7238), and the combined eight-species model similarly improved predictive accuracy (AUC = 0.8575). The consistent improvement of model performance through multi-species integration confirms its clinical advantage compared to single-biomarker diagnostics.

## 4 Discussion

This study presents a novel comparative framework characterising gut microbiota differences between BAP and HLAP, revealing distinct etiology-specific microbial signatures and identifying potential biomarkers for AP subtype classification. BAP and HLAP patients exhibited fundamentally different clinical phenotypes and metabolic profiles (Table 2). Host-microbiota correlation analyses revealed that these divergent host factors selectively shaped microbial community composition, creating distinct dysbiosis patterns (Figure 4). Specifically, HLAP patients exhibited more severe dysbiosis characterized by SCFA-producing bacteria depletion and pathogenic taxa enrichment. Given the critical roles of SCFA in gut-pancreas axis regulation, we further investigated whether these microbial alterations resulted in compromised SCFA biosynthetic capacity. Functional analysis revealed marked downregulation of key SCFA synthesis genes (*ackA*, *pta*, *buk*, *but*) in both AP subtypes (Figure 5), providing a mechanistic basis for targeted interventions. Finally, multi-species biomarker panels demonstrated robust diagnostic potential for AP subtype classification, suggesting clinical utility for precision medicine applications.

The more severe microbiota dysbiosis observed in HLAP patients, characterized by greater depletion of beneficial SCFA-producing bacteria (*Faecalibacterium*, *Bifidobacterium*) and enrichment of pathogenic taxa, correlates with the increased disease severity and poor prognosis in this subtype (Hu et al., 2021). As the most abundant genus among the top 20 and a key SCFA-producing genus, *Faecalibacterium* exhibited comprehensive correlations with clinical indicators in Spearman correlation analysis. This finding is consistent with its reported anti-inflammatory properties and intestinal barrier protective functions, highlighting its critical role in multiple pathophysiological processes (Lenoir et al., 2020). *Bifidobacterium*, recognized as a key beneficial genus with metabolic regulatory functions, showed notable alterations in AP patients (Li et al., 2022). Additionally, the expansion of opportunistic pathogens like *Escherichia-Shigella* and *Enterococcus* in HLAP patients, coupled with their positive correlations with inflammatory markers, highlights the distinct pathophysiological environments between BAP and HLAP subtypes (Zhang et al., 2025). These facultative anaerobes thrive in inflammatory environments and can translocate across compromised epithelial barriers, potentially contributing to the higher rates of systemic complications observed in HLAP (Li et al., 2023). The observed differences in microbiota composition reflect underlying mechanisms of gut-pancreas axis interactions (Yazici et al., 2023). These interactions are mediated through multiple pathways, including (1) inflammatory responses: the distinct pathophysiological mechanisms between biliary obstruction in BAP and lipotoxic injury in HLAP may cause inflammatory cascades by different signaling; (2) metabolic regulation: severe lipid dysregulation in HLAP patients may lead to different intestinal flora, or metabolites and toxins derived from microorganisms, enter the pancreatic microcirculation, further influencing disease progression (Han et al., 2023; Paik et al., 2022; Michaudel and Sokol, 2020).

Notably, alterations in key metabolites such as SCFAs potentially mediate gut-pancreas axis interactions (Ammer-Herrmenau et al., 2024). SCFAs have been proven to ameliorate bacterial translocation, a critical pathogenic mechanism in AP, by rebuilding gut flora and stabilizing the intestinal epithelial barrier (Yan et al., 2023). Moreover, SCFAs can suppress systemic inflammatory responses, improve the injured pancreas, and prevent and protect other organ dysfunctions (Li et al., 2020; He et al., 2020). Therefore, we analyzed four functional genes to assess SCFA biosynthetic capacity: *ackA* and *pta*, primarily involved in acetate synthesis; *buk* and *but* serving as two key genes in the butyrate-producing pathway (Huang et al., 2021). Our results revealed markedly reduced expression of acetate and butyrate synthesis genes (*ackA*, *pta*, *buk*, *but*) in AP patients. While our study identified impaired SCFA biosynthetic capacity associated with AP overall rather than subtype-specific changes, these findings provide rational therapeutic strategies for microbiota modulation in AP through probiotics, dietary interventions, or fecal transplantation.

Nonetheless, the study has limitations. Its cross-sectional design precludes causal inference, and whether dysbiosis precedes or results from AP onset remains to be established. While targeted qPCR validated key functional pathways, future metagenomics and metabolomics studies are needed to refine these findings. Additionally, age is not perfectly matched between groups in Table 2. However, a recent study with similar age differences (*p* = 0.004) successfully identified distinct microbiota signatures between HLAP and non-HLAP groups (Hu et al., 2021), supporting the validity of our analysis. In future studies, we will implement more stringent age-matching criteria in the HC group. Importantly, our correlation analysis demonstrates that age differences do not significantly confound our findings.

In conclusion, we establish a novel, etiology-specific framework linking gut microbial composition, function, and host phenotype in AP. This study advances the understanding of microbiota-driven mechanisms in AP pathogenesis and reveals distinct microbial signatures between BAP and HLAP subtypes. The significant discriminatory capacity of multi-species biomarker panels highlights their translational potential for precision diagnostics between BAP and HLAP. Future work should focus on refining key biomarkers and developing multiplex PCR assays for the simultaneous detection of these markers. Integration into microfluidic chip platforms could enable point-of-care testing, providing rapid subtype classification. This approach can help address current diagnostic challenges in borderline cases and support precision medicine in AP management.

## Data Availability

The data presented in this study are publicly available. This data can be found here: https://www.ncbi.nlm.nih.gov/sra, accession number PRJNA1330538.
